# Editorial: The Jilted Brain: Neglected Structures and Functions

**DOI:** 10.3389/fnsys.2021.716242

**Published:** 2021-08-11

**Authors:** Regina A. Mangieri, Kelly A. Allers, Natalie M. Zahr

**Affiliations:** ^1^Division of Pharmacology and Toxicology, College of Pharmacy, The University of Texas at Austin, Austin, TX, United States; ^2^Boehringer Ingelheim Pharma GmbH & Co. KG, Biberach, Germany; ^3^Department of Psychiatry and Behavioral Sciences, Stanford University School of Medicine, Stanford, CA, United States

**Keywords:** cerebellum, cuneiform nucleus, inferior colliculi, pedunculopontine nucleus, superior colliculi

The brain is an interconnected network of unique regions, differentiated by their location, the arrangement of distinct neurons and glia comprising them, the neurotransmitters or neuropeptides they produce, and myriad other features. Yet some brain regions (e.g., the basal ganglia) and some neurotransmitters (e.g., dopamine) remain at the forefront of neuroscience research and overshadow others. For example, Society for Neuroscience (SFN) 2019^*^ included 863 abstracts with the keywords “basal ganglia,” but only 390 abstracts for the keyword “cerebellum,” despite estimates that the cerebellum contains four times as many neurons as the cerebrum. Indisputably the entire brain and its many interactions are relevant for a better understanding of the brain in health and disease. The Research Topic “The Jilted Brain: Neglected Structures and Functions" was developed to highlight frequently overlooked brain regions or novel functions of better-studied brain regions. The unifying theme of the topic focused on generally under-reported brain structures or functions. The overarching goal was to collect the latest discoveries regarding “obscure” brain regions, thereby affirming the incalculable complexity of the brain.

The five articles included in the Research Topic are briefly discussed in order of first author's last name. In “The Inferior Colliculus in Alcoholism and Beyond,” Bordia and Zahr summarize findings from preclinical studies of alcohol exposure that consistently reveal volume reduction of the inferior colliculus in the context of other brain regions in the rodent brain. The authors posit that sensitivity of the inferior colliculi to alcohol exposure may be related to impaired mitochondrial function or oxidative stress. The authors hypothesize that the inferior colliculi have functions, possibly related to auditory gating, necessary for awareness of the external environment that may be impaired with chronic alcohol use. Their conclusions predict that alcoholism is associated with compromised synchronization of thalamocortical and pontocerebellar pathways due to inferior colliculi pathology.

In her report, “Not Part of The Temporal Lobe, But Still of Importance? Substantia Nigra and Subthalamic Nucleus in Epilepsy,” Bröer presents thoughtful and convincing data that seizure frequency and intensity may be attenuated by hindering conduction at the level of the basal ganglia circuitry. Bröer reviews the relevance of basal ganglia structures to treatment of seizure disorders by exploring preclinical research from the basal ganglia and epilepsy literature. Indeed, preclinical data suggest that disruption of seizure propagation in basal ganglia structures could be a successful means of treatment, and this notion is supported by the sparsely available clinical studies. Treatment strategies are not limited to standard pharmacotherapy but include surgical options as potential treatment approaches.

“Dissecting Brainstem Locomotor Circuits: Converging Evidence for Cuneiform Nucleus Stimulation” by Chang et al. was the most impactful of the articles submitted to this special issue with nearly 9,000 views and 600 downloads at the time this editorial was written. This interest is in stark contrast to only three abstracts published with the key word “cuneiform” at SFN 2019. In this Perspective, the authors summarize why the cholinergic pedunculopontine nucleus (PPN) was chosen as a target for deep brain stimulation (DBS) to treat gait disturbances in diseases such as Parkinson's and Supranuclear Palsy. The authors also proffer potential reasons why clinical trials targeting PPN failed and why the adjacent glutamatergic cuneiform nucleus is a superior target. They provide an excellent review of the anatomy of the relevant mesencephalic locomotor region. They hypothesize that successful DBS in the treatment of movement disorders will be due to orthodromic activation of efferent or afferent fibers of glutamatergic cuneiform neurons targeting reticulospinal neurons in the medulla that in turn excite spinal central pattern generators to facilitate gait.

The minireview presented by Miquel et al., “The Cerebellum on Cocaine,” updates perspectives first introduced by this group in 2009 and again in 2016 and reviews specific findings regarding the role of the cerebellum in cocaine-induced learning. Proposed mechanisms involved in persistent cocaine-related memories include increased granule cell activity and stabilization of perineuronal nets (which restrict neuronal plasticity) around Golgi interneurons in apical regions of the cerebellar cortex—a region that may be particularly relevant to drug addiction due to its relatively direct connectivity with the ventral tegmental area (*via* glutamatergic deep cerebellar nuclei). Further, individuals with substance use disorders exhibit a dysfunctional prefrontal-cerebellar pattern in which the cerebellum appears to be overactive during cognitive tasks that should recruit prefrontal resources, shifting the balance from model-based (goal-directed) to model-free (habit) systems.

In “The Hidden Brain: Uncovering Previously Overlooked Brain Regions by Employing Novel Preclinical Unbiased Network Approaches” by Simpson et al., the authors explain that neuroscientific inquiry has been hindered by the approach of focusing on brain regions implicated by prior work. They review recent technological advancements in preclinical whole-brain imaging, highlighting whole-brain tissue clearing methods and 4D functional ultrasound imaging. Further discussion includes the value of network-based, computational approaches for analyzing the vast amount of data generated by such technologies and their ability to identify previously overlooked but critical network “hubs” or “nodes” relevant to normal and pathological brain function.

In summary, the included articles demonstrate that novel cell-specific tools, such as optogenetics, advanced viral tracing, and computational methods, have potentiated scientific inquiry, especially into brain regions that have been historically difficult to access and investigate. Moving forward, it is essential that scientists maintain a disinterested view of the data generated by such novel technologies and the courage to challenge the status quo regarding the relevance of underrepresented regions to a better understanding of brain structure and function in health and disease.

^*^14,013 abstracts accepted for SFN 2019

Abstract numbers listed below from online meeting planner: https://www.abstractsonline.com/pp8/#!/7883

1,941 Hippocampus

1,770 Temporal lobe

1,428 Pedunculopontine Nucleus

863 Basal Ganglia (BG)

881 Dopamine

736 BG Striatum

444 BG Nucleus Accumbens

390 Cerebellum

290 Inferior Colliculus

264 BG Substantia Nigra

98 BG Caudate

76 BG Putamen

68 BG Subthalamic Nucleus

68 Superior Colliculus

58 BG Globus Pallidus

3 Cuneiform Nucleus.

## Author Contributions

All authors summarized assigned manuscripts and reviewed the editorial.

## Conflict of Interest

KAA was employed by the company Boehringer Ingelheim Pharma GmbH & Co. KG. The remaining authors declare that the research was conducted in the absence of any commercial or financial relationships that could be construed as a potential conflict of interest.

## Publisher's Note

All claims expressed in this article are solely those of the authors and do not necessarily represent those of their affiliated organizations, or those of the publisher, the editors and the reviewers. Any product that may be evaluated in this article, or claim that may be made by its manufacturer, is not guaranteed or endorsed by the publisher.

